# Time, trust, and relationships: Creating a culture of community engagement to advance translational research through resource allocation, modeling, and recognition

**DOI:** 10.1017/cts.2025.10131

**Published:** 2025-09-26

**Authors:** Linda Susan Sprague Martinez, Riana C. Howard, Melanie Rocco, Jennifer Pamphil, Deborah Chassler, Astraea Augsberger, Tracy A. Battaglia, Rebecca Lobb

**Affiliations:** 1 Health Disparities Institute, UConn Health, Hartford, USA; 2 Boston University School of Social Work, Boston, MA, USA; 3 Boston University Clinical Translational Science Institute, Boston, MA, USA; 4 Boston University Medical Campus, Boston, MA, USA

**Keywords:** Community research partnerships, seed grants, incentivizing community engagement, translational research

## Abstract

Advancing community engagement and participatory research approaches necessitates shifting cultural norms. The paper describes a program designed to explicitly embed and reinforce a culture of engagement through resource allocation, modeling, and recognition that was initiated by a Clinical and Translational Science Institute Community Engagement Program CE Program. Resources were allocated to the relationship development process between researchers and community partners. Funded partnerships were provided with guidance to support the equitable distribution of resources. Partnerships received additional reinforcement through participation in a learning collaborative, intended to support community partnership development, model best practices in community engagement and to build a network of community engaged, and participatory researchers at the institution. Investigators reported the learning collaborative “gave them permission” to focus on the process. Overall, lessons learned indicate embedding and reinforcing practices that center relationship and reward time spent building partnerships is a promising strategy to buffer against cultural norms that favor outcomes and over process.

## The research scholars partnership grant: Creating a culture of community engagement to advance translational research through resource allocation, modeling, and recognition

Translational research is designed to move evidence generated in laboratory, clinical, and community settings into interventions that improve the health of people and communities [1,2]. It has been established in the literature that community engagement is a key factor for advancing translational research, however, the uptake of community engaged and participatory approaches remains low [3,4]. The limited adoption of community engaged and participatory approaches have been associated with multiple factors including trust and time [5–7]; which are interconnected given establishing trust requires time [6,8]. Trust, mistrust, and trust worthiness in community engagement are frequently interrogated in the literature [9–12]. References to time have been less robust and primarily focus on the time intensive nature of community engaged and participatory approaches [13]. Critical reflections on time point to the ways in which it is shaped by cultural norms such as white supremacy and the political economy [14–17].

White supremacy is the dominant world view ideology in the United States; it was established during the age of enlightenment to uphold the institution of slavery [18–20]. Under white supremacy, value, expertise, and power are assigned to whiteness [21,22]. This influences both trust and time because ideology shapes social norms, which inform policy and in turn organizational practice. Sense of urgency has been described as a core tenant of white supremacy which values outcomes over process and focuses on individual as opposed to collective processes, which are more time consuming. A constant sense of urgency can break down trust and expose power imbalances between researchers and community partners. In addition, it can disrupt inclusive consensus building processes, because time constraints favor either/or thinking and top-down decision-making. This can limit participation, moreover, needing to do things at a fast pace doesn’t facilitate the relationship development and trust building needed for co-creation and collaboration [9,23,24]. Indeed, the structure of research funding and funder expectations can exacerbate sense of urgency. Short timelines instituted by funders and or academic institutions, often fail to take into consideration the time needed for collaborative processes. This is reinforced by a culture of individualism. Urgency undermines researchers, institutions and communities when the importance of outcomes overshadows that of processes, such as relationship development and trust building.

Taking the time to build relationships necessitates a recognition that conceptualizations of time vary. As we engage with communities, it is critical for researchers to honor the variation in how time is understood at the individual, interpersonal, community, and societal levels [25,26]. To illustrate this, Fischer, Reuber, Hababou, Johnson and Lee (1997) write that time…

*…must be viewed not as a linear, uncontrollable fact of life but as a variable, socially constructed feature of enacted organizational realities. This perspective posits that time is experienced inter-subjectively - and distinctively - within particular groups, communities, and organizations (Giddens, 1984; Clarke, 1985)* [27].

Time is shaped by cultural norms as well as maintained and reproduced by at various levels which can shape organizational practice. As such, advancing community engagement and participatory research approaches necessitates shifting culture norms as they relate to time and more specifically, the sense of urgency embedded in academic research processes.

We present a Boston University Clinical and Translational Science Institute Community Engagement Program (CE Program) program designed to create a culture that centers time for relationship development and engagement. In the spring of 2022, the CE Program launched the Research Scholars Partnership Grant program, with the intention of funding researchers and community members to facilitate the development of community-research partnerships. In this paper, we provide a brief background on organizational culture and the ways it impacts community engagement and shapes researcher behavior followed by a detailed description of the Research Scholars Partnership Grant and program outcomes. We conclude by discussing our findings and lessons in the context of the literature.

## Organizational culture and community engagement

Organizational culture shapes how members of an organization learn behavioral expectations and interact [28]. Given its multidimensional complexity, definitions of culture vary. However, there is agreement that culture is learned and shared through interactions and guides societal life as members develop a shared set of common beliefs [29]. To that end, organizational culture has been defined as behaviors, values, perspectives, and expectations that are shared by organizational participants [28,30]. In the case of academic research institutions, understanding culture is an important component of efforts to advance community engagement because culture shapes and reinforces behaviors.

**Figure 1.**
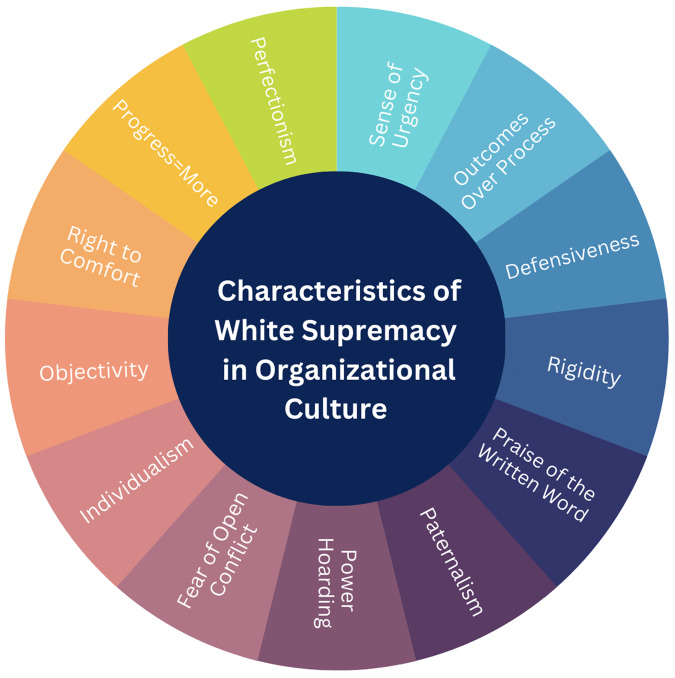
White supremacy characteristics in organizations [18].

Academic research institutions are embedded in a broader sociopolitical landscape, and as noted, the white supremacy is the dominant ideology that shapes the sociopolitical landscape [31,32]. Thus, white supremacy also shapes organizational practice. Okun and others have described characteristics of white supremacy at the organizational level (see Figure 1) [33].

These ideas are embedded and intertwined with organizational culture and in turn the interactions and behaviors among organizational members as well as how members interact with people outside the organization. Notably, in the context of white supremacy outcomes are favored over processes to develop relationships and as such there is a sense of urgency to advance production. This is particularly challenging for community engagement and participatory research approaches, which rely heavily on processes which require time to build trusting relationships across cultural boundaries of organizations and communities. Not surprisingly, herein lies the principal contradiction, as community engaged, and participatory approaches are rooted in theories of collectivism which runs counter to racial capitalism fueled by an ideology of white supremacy, which centers individualism.

Shifting organizational culture is complex because culture is not tangible and the longer we are in a culture the more unaware of it we become [31]. The literature indicates there are primary cultural embedding mechanisms and secondary reinforcement or articulation mechanisms [28]. Primary mechanisms are what leaders pay attention to. For example, what they choose to measure and reward, how they allocate resources, and what behaviors they model. Secondary reinforcement and articulation mechanisms include organizational design as well as systems, procedures, and values statements [28]. Thus, promoting community engaged and participatory research approaches necessitates leveraging mechanisms to shift culture [34,35]. For example, short-term pilot funding that does not financially support the time it takes to develop academic community relationships and a focus on research outcomes over process enshrined in the materials and procedures as well as in our overall institutional system of rewards will contribute little to developing a culture of community engagement.

With the Research Scholars Partnership Grant program, we sought to explicitly create time for partnership development as well as to reinforce a culture of engagement through resource allocation, modeling, and recognition. We reviewed the number of community engaged and participatory applications being submitted and explored barriers to community engaged pilots as a team. One change that emerged through discussions was the short timeline associated with the pilot grants program does not allow time for partnership development. Thus, we sought to allocate resources specifically to the relationship development process between researcher and community partners and provided guidance to support the equitable distribution of resources. Partnerships were required to participate in a three-part learning collaborative, during which we focused explicitly on modeling best practices in community engaged and participatory research through co-learning. Finally, we recognized and promoted the work of each partnership by announcing their award in CTSI mailings and updates as well as on our website. We also featured each partnership during our culture of community engagement speaker series event.

## The research scholars partnership grant

In May 2022, we released the request for proposals for the Research Partnership Scholars Grant program. The primary objective of the program was to support the development of community-academic partnerships. It was expected that dedicating time to partnership development would eventually lead to an increase in the number of community engaged and participatory applications for extramural funding focused on advancing health equity. The grant itself allowed for nine months of funding up to $10,000 and was open to BU researchers and community partners, with either being the primary applicant.

The CTSI Community Engagement program (CTSI CE) advertised the request for applications broadly across the university and through community partner networks, including the CTSI CE program community advisory board, which focuses on advancing health equity research [36]. Grant application guidance was provided on the program website and a preapplication webinar with a question-and-answer period was held. In addition, community engagement program staff provided technical assistance consultations to support partnerships in developing and completing the application and to connect interested researchers with community partners and vice versa.

**Figure 2.**
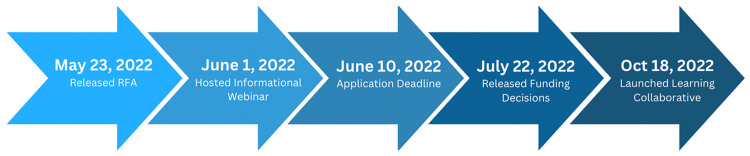
Timeline.

As detailed in Figure 2: Timeline, once the application was released in early May 2022, applicants had until June 10, 2022, to complete and submit their application. Applications were then reviewed by selected reviewers and applicants were notified of the final decisions on July 22, 2022. Upon notification of application decisions, applicants were then invited to attend the three-part mandatory learning collaboratives that took place in Fall of 2022. Details on each step of the process are provided in the sections that follow.

## Application guidelines

Community and academic researchers were required to submit the three-page application as co-Principal Investigators. Additional required materials included a biosketch or resume for each partner, as well as a budget and budget justification. As one of the intentions behind this grant was to build faculty capacity for and interest in community engaged and participatory research approaches, academic researchers were required to be BU full or part-time faculty, and community partner researchers had to work in a community, grassroots or faith-based organization and could also be an independent contractor with lived experience within the population of interest. Research experience was not required for the community investigator, but funded community partners were required to participate in the three-part learning collaborative.

Applicants were required to allocate at least 50% of the grant budget to the community partner investigators. We held a webinar, office hours, and individual consultations for applicants to support proposal development and budgeting. Activities eligible to be funded as part of the grant were focused on relationship building. This included developing new relationships or strengthening an existing partnership. Additional allowable expenses included time to explore shared research interests; capacity building for team members; advisory boards; and participatory planning activities; as well as preliminary data collection, analysis, and dissemination.

## Application review and selection

We received seven applications in response to the RFA. Review of the submitted applications for this program was sectioned into three phases: (1) community and academic review; (2) internal CTSI CE review; and (3) CTSI leadership review of funding decisions. The first phase of review was conducted by volunteer community and academic reviewers. One academic and one community reviewer were assigned to independently evaluate each grant application. Community reviewers received a $25 gift card for taking part in the review. Reviewers were asked to assess the responsiveness to the RFA based on five criteria: (1) aims of partnership, (2) background and significance of the partnership, (3) strategy and partnership development plan, (4) dissemination plan and timeline, (5) overall project focus on health equity. Questions were associated with each criterion to clarify the definition of the criterion and prompt reviewers’ justification of the score associated with the criterion. The scores for each criterion reflected the National Institutes of Health 9-point scale (1–3 high strength, 4–6 medium strength, 7–9 low strength). Scoring and feedback by academic and community reviewers were then synthesized and ranked for review by the internal CTSI CE team.

The CE CTSI team met to consider the reviewers’ scores and discuss the responsiveness of the applications to the RFA. The average application score for the community-academic reviewer dyad ranged from 2.0 to 6.0. Overall, there was consistency between community and academic reviewers’ scores. Of the seven applications, the difference in scores was one point or less for four applications, two points for two applications, and five points for one application. Applications that were not responsive to the award notice including the potential activities of the RFA, allowable costs, or did not demonstrate collaborative involvement from the community partner were removed from consideration. The applications with reviewer dyad scores that differed by two points, or more were discussed by the CE CTSI team to reconcile the discrepancy. The CE CTSI team’s final recommendation, with justification for funding three racially and ethnically diverse partnerships, were submitted to the CTSI leaders, and received their approval (see Table 1). Table 1 describes the three funded partnerships.

**Table 1.** Funded partnerships

Partnership Descriptions
The first partnership that was awarded included a partnership among Boston University, Becca Schmill Foundation, and UMass Memorial. Shared interests between the researchers and community partners included youth overdose prevention. Group goals also included relationship building and learning more about the development of community and youth boards.
The second partnership that was awarded included a partnership among Boston University and De Novo. Shared interests between the researchers and community partners included improving mental health of immigrants, refugees, and asylum-seekers in Boston. Group goals included relationship development, learning more about shared decision-making models and collaborative research approaches.
The third partnership that was awarded included a partnership among Boston University and Patang Hospital in Accra, Ghana. Shared interests between the researchers and community partners included improving care for women and girls with substance use disorder in Accra, Ghana. Group goals also included learning critical skills for group work, shared decision-making and collaborative research.

## Learning collaborative

Partnerships were required to participate in a three-part learning collaborative, which was intended to support community partnership development, model best practices in community engagement, and to build a network of community engaged and participatory researchers at the institution. The learning collaborative was facilitated by the CE Program Co-Director, a BU faculty member with expertise in community engaged and participatory research approaches and a social work doctoral student. Overall, the learning collaborative was designed to provide time for processes including partnership development, identifying shared research interests, and collectively designing research.

Learning Collaborative content was informed by the literature as well as by data collected from partnerships. Data, specifically, was used to determine variation among participants in their familiarity and confidence with Community Engaged Research (CEnR). The learning collaborative focused on building a shared understanding among partnerships of the foundations of community-based participatory research [5,37], the spectrum of community engaged and participatory research approaches [38–40], the stages of group development [38], shared decision-making practices, and research planning processes [41–44].

Sessions were hosted on Zoom and each lasted 90 minutes. The sessions were interactive, and to facilitate partnership development they involved information sharing, team activities to explore concepts of power, communication, expectations, which influenced their overall team planning. Each session followed a similar format. The facilitators who both identified as Black women welcomed the three partnerships. reviewed session goals, conducted a group check in as well as a warm-up activity, and reviewed the group commitments. The remainder of each session involved full group learning and discussion, small group and pair activities, and application of the content through partnership team planning. During sessions participants were encouraged to discuss and grapple with the content, as well as to share examples from their own practice, recognizing the expertise that each member brought to the group. At the end of each session “plus/delta” was used to solicit what participants enjoyed about the session and to solicit changes for the following session. Between sessions, the facilitator shared reading and resources based on the questions and discussion that came up in during the session.

The primary goal of the first session was to develop a shared understanding among participants with respect to the foundations of community engaged and participatory approaches to research, the principles of CBPR, the spectrum of community engaged and participatory approaches, and the emergence of CEnR and participatory approaches in the context of health research in the United States. This session culminated with research teams reflecting on their goals and expectations as well as the strengths and assets they bring to the team as individuals and organizations.

Session two was focused on partnership development. Content covered during the session included positionality, the phases of group development and group decision-making models. During the warm-up, participants reflected in mixed team pairs on “…a time they had to make a decision as part of a group.” They were asked to describe in one word… “What it felt like? What was easy? What was hard?” They then shared themes that emerged during their pair share with the larger group. This was followed by a facilitated discussion on trust and the factors that facilitate trust building based on participant experience. A paired activity was then used to allow participants to shared different elements of their identity with one another. This allowed participants to explore deeper connections with one another and was followed by a large group discussion of position and specifically, how position influences how one thinks and behaves. Through this discussion participants reflected on their own experiences and positionality and hypothesized about how it might influence their partnership and work together. At this point the concept of struggle was introduced with a quote for the group to reflect on.

*We struggle for the sake of building deeper unity, that we are honest and direct while holding compassion, that we each take responsibility for our own feelings and actions and seek deeper understanding by asking questions* [44].

The group discussed struggle as necessary for growth and learning in the context of partnership as well as group development. The session culminated with teams working in breakout rooms to discuss the relationships within the partnership. This involved thinking about “how they would be together.” More specifically, “how they would work through differences of opinions, have difficult conversations and solve problems.”

**Figure 3.**
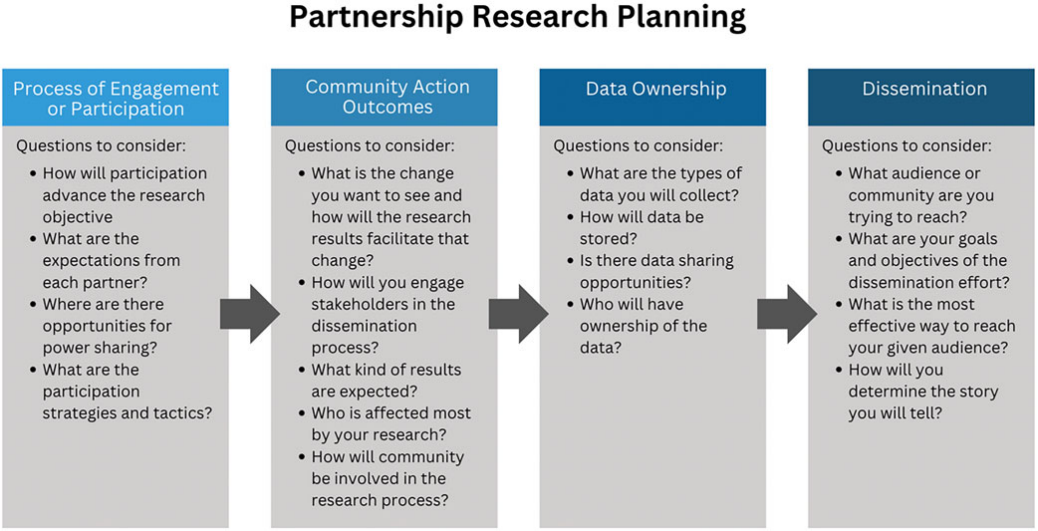
Partnership research planning.

The final session was focused on research plan development. The facilitators introduced a slide with a series of considerations related to the process of engagement or participation, community action outcomes, data ownership, and dissemination (see Figure 3).

Groups worked for 45 minutes on this individual research team planning in breakout rooms. At the end of the session, they came back to the larger group and discussed themes related to decision-making, power-sharing, community action and change, data sharing, and dissemination. Consensus best describes how teams were approaching decision-making, although it was not the term they used. They described their process as one of laying out the options, getting input from everyone on the team, laying out the pros and cons, and then moving forward in a direction that all members of the team could get behind. The session culminated with an overall reflection on the learning collaborative process using a plus/delta activity.

## Evaluation

The CE Program administered pre-post-evaluation surveys to all three funded partnerships (five researchers and 8 community members), via an email survey link, to inform the development of the learning collaborative and evaluation of the Research Scholars Partnership Grant program. Surveys were distributed after the funding notice was received (pre) and after the final learning collaborative session (post). Data were recorded and managed in Redcap [45] and then exported to Microsoft excel for analysis. Descriptive statistics were calculated and open-ended items were analyzed thematically by question.

The three closed-ended survey questions, administered at baseline, were used to inform learning collaborative content.

- How familiar are you with the CBPR principles?
- How confident are you in engaging in CEnR?
- How familiar are you with the theoretical foundations of CEnR?

The response options ranged from 1 (low) to 5 (high). The mean responses to these survey items at baseline were 2.2, 2.7, and 2, respectively.

The three closed-ended survey questions administered post participation, were used to evaluate satisfaction of the learning collaboratives and to explore the impact of the learning collaborative in creating time for partnership development and collective planning.

- How satisfied were you with this learning collaborative?
- How effective were the facilitators in this learning collaborative?
- How relevant was the LC content for your research collaboration?

The response options ranged from 1 (low) to 5 (high). Participants noted a mean average of 4.8 in satisfaction, a 5 for effectiveness in how the learning collaboratives were facilitated, and a 4.8 stating how relevant the learning collaborative was to their research collaborative.

A fourth closed-ended question was administered post participation to evaluate for an increase in confidence in engaging in CEnR post participation of the learning collaboratives.

- After attending the LC, how confident are you in engaging in CEnR?

The response options ranged from 1 (low) to 5 (high). Over the course of the learning collaborative participants’ confidence in engaging in CEnR increased from a mean of 2.7 (scale 1–5) to 4.3.

At the end of the learning collaborative, participants completed a post survey regarding their experience participating in the learning collaborative. On a scale of 1 (low) to 5 (high), mean scores were high for satisfaction, effectiveness in how the learning collaborative was facilitated, and relevance to participant research collaborations: 4.8, 5, and 4.8, respectively. Overall, researchers and community partners spoke highly about the learning collaborative and appreciated having the time to reflect and plan as teams. In some cases, partners indicated wanting more sessions together to plan with their team as well as continued co-learning with other teams.

*I don’t think this project would have happened at all without this LC [learning collaborative] and grant.* Researcher

*…we now have a framework (and experience) to talk about community engagement in research. We also have a network of others doing this work to request help when needed.* Researcher

*I wish the learning had more than 3 sessions maybe 5.* Community partner

Responses indicated researchers and community partners felt more confident.

*It [the learning collaborative] gave me more confidence to pursue community-engaged projects.* Researcher

*…for the first time, I am feeling excited about doing research due to the participatory and community focused nature of it. I could definitely see myself continuing with this type of research.* Community partner

*…it [the learning collaborative] has equipped me to understand that the members of the community are also co-partners in the research process”* Community partner

Meanwhile, researchers shared reflections on the benefits of the paying attention to process and the importance of both connecting with partners and approaching relationship development with intention.

*I am being more thoughtful about ways that partners and I can agree on the boundaries of the partnership at the outset.* Researcher

*I am paying more attention to the process.* Researcher

*That I would be willing to be more engaging and accept other people’s opinions even if I don’t agree with it. And all should feel at ease to discuss all issues relating to the research.* Researcher

*I can engage community partners better … giving the opportunity to feel and understand the emotions of partners on topics they feel uncomfortable with.* Researcher

## Discussion

Direct funding for the development of community-research partnerships is critical, but it is not enough on its own to foster successful community engaged and participatory research. Trust and relationship building are critical, and these processes require time to build mutual trust that can lead to a shared vision, collaborative decision-making, and resource sharing – something that is often undervalued in research funding models. However, research funding is generally focused on outcomes rather than process and funding alone does not lay the foundation for co-learning, power-sharing, and joint ownership. The pressure to achieve quick outcomes, driven by institutional urgency and external deadlines, can undermine the long-term investment needed to establish meaningful partnerships. As discussed, urgency in research settings – rooted in white supremacy culture – prioritizes speed and outcomes over process, creating a power imbalance that hinders genuine collaboration. The goal of the partnership research scholars grant program was to embed and reinforce a culture of community engagement through funding, training, and recognition to demonstrate that the processes associated with community engagement are valued.

There has been an increase in grants to fund the development of research partnerships over the past 15 years [46–50]. Described as seed and pilot grants; these funding mechanisms are designed to catalyze community-based participatory research. In some cases, grants are made directly to community partners as the primary recipient. For example, the Harvard Catalyst and the Atlanta Clinical and Translational Science Institute provide funds directly to community-based organizations to increase their research infrastructure [49,50]. Other models funded applications from both community and academic leads [47,51]. Although, most grants range in size ($2,000–$30,000) they share common elements including a structured application, required resource sharing, a project-based focus, and an application review by community members and researchers as well as technical assistance and or training [46,47,49–51]. This literature that describes research partnership development grants highlights the need for time to focus on processes to support the partnership [46,50]. and access to information and technical support to learn which processes to use [51].

Consistent with the literature on community-based participatory research, we found that partners appreciated having funding opportunities that enable time dedicated to come together to plan and to think about the “how” of community engagement. Our program emphasizes the importance of time – not just for the logistics of research, but for the relational work necessary to develop shared goals and collaborative decision-making. Researchers described the grant and learning collaborative as “giving them permission” to make time for relationship building. They had time to meet, time to reflect and time to plan. The relationship building process was prioritized and centered as the primary outcome. This was seen by teams as a cultural shift and very different from the norm in which the research deliverables are centered and prioritized.

We also found that others implementing seed grant programs focused on CBPR often offered training for community and academic partners, consistent with these reports we found that grantees appreciated the opportunity to learn and apply skills in real time [46]. There are limited in-depth descriptions of the training programs offered. In general, the training content focused on CE skills, IRB, administrative issues, and research methods [46,47,51]. The training formats included sessions, seminars, forums, and mentoring [46,47,51].

In contrast to other partnership grants that provided training, our program incorporates a learning collaborative approach focused on developing CE skills. Collaborative learning was further enhanced through brainstorming with other research partnerships and exchanging best practices and lessons learned [46]. Through the learning collaborative participants developed and increased their familiarity with community engaged and participatory research. Researchers developed relationships with community members and through applied co-learning which substantially increased their confidence with community engaged and participatory research.

Overall, partners found the learning collaborative to be quite effective as a model for creating time and space to focus on the mechanics of partnership development. Learning collaboratives are synonymous with communities of practice and learning communities [52]. They are defined in the literature as “groups of people who share a concern or a passion for something they do and learn how to do it better as they interact regularly” (p.1) [53]. This is what makes the learning collaborative model the ideal learning approach for partnership grant trainings. Inherent in the model is the recognition that each member brings with them practical expertise to share as such the facilitator is just one of many experts in the space. Group discussions and team-based activities allow for real world application. As members share their respective expertise and learn from one another relationship building occurs [53]. To that end the learning collaborative is designed to be sustained overtime as members develop shared resources and experiences [53].

This work is not without limitation. These are early findings and with only three partnerships, the sample was small, and the results are not statistically significant. To date the results have been positive and we expect to learn more over time as we bring in more partnerships. It is also hard to say if the results are a direct result of the learning collaborative content of the relationships developed between the facilitators and the participants. In addition, we would be remiss not to mention our own challenges with time pressure and the ways in which early on we passed these challenges on to applicants. Namely, our short application window. However, we acknowledged this in sessions and used it as an example of how structures shape our behavior. Through this pilot we learned important lessons with respect to intentional approaches for catalyzing partnership development. More specifically, we learned that embedding and reinforcing practices that center relationships and reward time spent building partnerships is a promising strategy to buffer against cultural norms that favor outcomes and over process.

## Conclusion

These findings indicate prioritizing relationship development through funding, paired with a learning collaborative, may increase researcher familiarity and confidence with community engaged and participatory research approaches. This mode can “give permission” to investigators to invest time in the process of relationship development, which is critical to developing a shared research agenda. Like others we encourage funders to build-in time and funds for partnership development.[51].

## References

[1] Richmond A , Aguilar-Gaxiola S , Perez-Stable EJ et al. Proceedings of the 2017 advancing the science of community engaged research (CEnR) conference. BMC Proceedings. 2019;13:3–21. doi: 10.1186/s12919-019-0164-y. 31019549 PMC6474049

[2] Stallings SC , Boyer AP , Joosten YA , et al. A taxonomy of impacts on clinical and translational research from community stakeholder engagement. Health expectations : an international journal of public participation in health care and health policy. 2019;22:731–742. doi: 10.1111/hex.12937. 31321849 PMC6737764

[3] Freeman E , Seifer SD , Stupak M , Martinez LS. Community engagement in the CTSA program: stakeholder responses from a national Delphi Process. Clin Transl Sci. 2014;7:191–195. doi: 10.1111/cts.12158. 24841362 PMC5350819

[4] Sprague Martinez L , Chassler D , Lobb R , Hakim D , Pamphile J , Battaglia TA. A discussion among deans on advancing community engaged research. Clin Transl Sci. 2023;16:557–563. doi: 10.1111/cts.13478. 36707736 PMC10087072

[5] Israel BA , Parker EA , Rowe Z , et al. Community-based participatory research: lessons learned from the centers for children’s environmental health and disease prevention research. Environ Health Persp. 2005;113:1463–1471. doi: 10.1289/ehp.7675. PMC1281296 16203263

[6] Metzler MM , Higgins DL , Beeker CG , et al. Addressing urban health in Detroit, New York City, and Seattle through community-based participatory research partnerships. Am J Public Health (1971). 2003;93:803–811. doi: 10.2105/AJPH.93.5.803. PMC1447843 12721148

[7] Sprague Martinez L , Carolan K , O.’Donnell A , Diaz Y , Freeman ER. Community engagement in patient-centered outcomes research: benefits, barriers, and measurement. J Clin Transl Sci. 2018;2:371–376. doi: 10.1017/cts.2018.341. 31404157 PMC6676439

[8] Palmer-Wackerly AL , Krok JL , Dailey PM , Kight L , Krieger JL. Community engagement as a process and an outcome of developing culturally grounded health communication interventions: an example from the DECIDE project. Am J Commun Psychol. 2014;53:261–274. doi: 10.1007/s10464-013-9615-1. 24567052

[9] Lansing AE , Romero NJ , Siantz E , et al. Building trust: leadership reflections on community empowerment and engagement in a large urban initiative. BMC Public Health. 2023;23:1252–1252. doi: 10.1186/s12889-023-15860-z. 37380973 PMC10304359

[10] Mullins CD , Tanveer S , Graham G , Baquet CR. Advancing community-engaged research: increasing trustworthiness within community-academic partnerships. J Comp Effect Res. 2020;9:751–753. doi: 10.2217/cer-2020-0096. 32815743

[11] Smirnoff M , Wilets I , Ragin DF , et al. A paradigm for understanding trust and mistrust in medical research: the community VOICES study. AJOB Empirical Bioethics. 2018;9:39–47. doi: 10.1080/23294515.2018.1432718. 29368998 PMC6092744

[12] Wilkins CH. Effective engagement requires trust and being trustworthy. Med Care. 2018;56:S6–S8. doi: 10.1097/MLR.0000000000000953. 30015725 PMC6143205

[13] Yonas MA , Jones N , Eng E , et al. The art and science of integrating undoing racism with CBPR: challenges of pursuing NIH funding to investigate cancer care and racial equity. J Urban Health. 2006;83:1004–1012. doi: 10.1007/s11524-006-9114-x. 17072760 PMC3261297

[14] Banaji MR , Fiske ST , Massey DS. Systemic racism: individuals and interactions, institutions and society. Cognitive Research: Principles and Implications. 2021;6:82–82. doi: 10.1186/s41235-021-00349-3. 34931287 PMC8688641

[15] Crenshaw KW . Mapping the margins: Intersectionality, identity politics, and violence against women of color. In: The Public Nature of Private Violence. Routledge, 2013: 93–118.

[16] Lawrence S , Hylton K. Critical race theory, methodology, and semiotics: the analytical utility of a “Race” conscious approach for visual qualitative research. Cultural Studies, Critical Methodologies. 2022;22:255–265. doi: 10.1177/15327086221081829.

[17] Vue R , Ly KT , Porter T , Aguilar AA. Feeling the threat of race in education: exploring the cultural politics of emotions in CRT-ban political discourses. Educ Eval Policy An. 2024;46:222–248. doi: 10.3102/01623737231221155.

[18] DiTomaso N. The invention of race and the persistence of racial hierarchy: white privilege, white supremacy, and white colorblindness. Social and Personality Psychology Compass. 2024;18:e12953. doi: 10.1111/spc3.12953.

[19] Eyerman R. White Consciousness from Colonization to the Civil War. Oxford University Press, 2022.

[20] Thiaw I , Mack DL. Atlantic slavery and the making of the modern world: experiences, representations, and legacies: an introduction to supplement 22. Curr Anthropol. 2020;61:S145–S158. doi: 10.1086/709830.

[21] Drustrup D , Liu WM , Rigg T , Davis K. Investigating the white racial equilibrium and the power-maintenance of whiteness. Anal Soc Iss Pub Pol. 2022;22:961–988. doi: 10.1111/asap.12321.

[22] Sow MJ . Whiteness as contract. Wash. & Lee L. Rev. 2021;78:1803.

[23] Bien-Gund S , Elrowmeim S. Building trust and cultivating connection, leadership, and empowerment in a community-based program. New Directions for Adult and Continuing Education. 2019;2019:59–69. doi: 10.1002/ace.20356.

[24] Krczal E , Behrens DA. Trust-building in temporary public health partnerships: a qualitative study of the partnership formation process of a covid-19 test, trace and protect service. Bmc Health Serv Res. 2024;24:467–467. doi: 10.1186/s12913-024-10930-3. 38614970 PMC11015697

[25] Hardcastle B , Byrnes D , Bartlett A , Denton P , Walsh PR. The Ecology of Human Development-Experiments by Nature and Design by Urie Bronfenbrenner. Cambridge, Ma: Harvard University Press, 1979: 330. $16.50. Taylor & Francis Group; 1981. p. 117–123.

[26] Sprague Martinez L , Negrón R , Brinkerhoff CA , et al. Project aquiLá: community-engaged planning to explore the relationship between culture and health. Progress in Community Health Partnerships. 2023;17:307–317. doi: 10.1353/cpr.2023.a900211. 37462559

[27] Fischer E , Reuber AR , Hababou M , Johnson W , Lee S. The role of socially constructed temporal perspectives in the emergence of rapid-growth firms. Entrep Theory Pract. 1998;22:13–30. doi: 10.1177/104225879802200203.

[28] Bartholomew LK , Parcel GS , Kok G , Gottlieb NH , Fernandez ME. Planning health promotion programs: Intervention mapping approach. 3rd ed. Jossey-Bass, 2011: 154–160.

[29] Kottak CP , Kottak CP . Mirror for Humanity: A Concise Introduction to Cultural Anthropology. New York: McGraw-Hill, 1996.

[30] Bellot J. Defining and assessing organizational culture. Nursing Forum (Hillsdale). 2011;46:29–37. doi: 10.1111/j.1744-6198.2010.00207.x. 21306393

[31] dRworks. Dismantlingracism.org. (https://www.dismantlingracism.org/) Accessed January 14, 2022.

[32] Belkin Martinez D , Hamilton G , Toraif N . The learning catalog: Understanding structural & institutional racism. Boston University School of Social Work, The Network for Professional Education. (https://thenetwork.bu.edu/offering/understanding-structural-institutional-racism/) Accessed January 14, 2022.

[33] Okun T. White supremacy culture characteristics. (https://www.whitesupremacyculture.info/characteristics.html) Accessed January 14, 2022.

[34] Kania J , Williams J , Schmitz P , Brady S , Kramer M , Juster JS. Centering equity in collective impact. Policy & Practice. 2022;80:12–19.

[35] National Cancer Institute. (2005). Theory at a glance: A guide for health promotion practice (NIH Publication No. 05-3896). U.S. Department of Health and Human Services, National Institutes of Health, Besthsda, MD. (https://cancercontrol.cancer.gov/sites/default/files/2020-06/theory.pdf) Accessed January 14, 2022.

[36] Institute BUCTS. Community-academic partnerships. (https://www.bu.edu/ctsi/community-engagement/community-academic-partnerships/) Accessed January 14, 2022.

[37] Israel BA , Lichtenstein R , Lantz P , et al. The detroit community-academic urban research center: development, implementation, and evaluation. J Public Health Man Pract. 2001;7:1–19. doi: 10.1097/00124784-200107050-00003. 11680026

[38] Agency for Toxic Substances and Disease Registry (ATSDR). Figure 1.1. Community engagement continuum | principles of community engagement. (https://www.atsdr.cdc.gov/communityengagement/community-engagement-continuum.html) Accessed January 14, 2022.

[39] Key KD , Furr-Holden D , Lewis EY , et al. The continuum of community engagement in research: a roadmap for understanding and assessing progress. Progress in Community Health Partnerships. 2019;13:427–434. doi: 10.1353/cpr.2019.0064. 31866597

[40] Schlechter CR , Del Fiol G , Lam CY , et al. Application of community – engaged dissemination and implementation science to improve health equity. Preventive Medicine Reports. 2021;24:101620–101620. doi: 10.1016/j.pmedr.2021.101620. 34976676 PMC8684008

[41] Becker AB , Israel BA , Allen AJ. Strategies and techniques for effective group process in CBPR partnerships. Jossey-Bass, 2005.

[42] Oetzel JG , Boursaw B , Magarati M , et al. Exploring theoretical mechanisms of community-engaged research: a multilevel cross-sectional national study of structural and relational practices in community-academic partnerships. Int J Equity Health. 2022;21:59–59. doi: 10.1186/s12939-022-01663-y. 35501798 PMC9063068

[43] Sanchez-Youngman S , Boursaw B , Oetzel J , et al. Structural community governance: importance for community-academic research partnerships. Am J Commun Psychol. 2021;67:271–283. doi: 10.1002/ajcp.12505. 33890308

[44] Wallerstein N , Oetzel J , Duran B , Tafoya G , Belone L , Rae R. What predicts outcomes in CBPR. Community-based participatory research for health. Jossey-Bass, 2008: 371–392.

[45] Harris PA , Taylor R , Thielke R , Payne J , Gonzalez N , Conde JG. Research electronic data capture (REDCap)—A metadata-driven methodology and workflow process for providing translational research informatics support. J Biomed Inform. 2009;42:377–381. doi: 10.1016/j.jbi.2008.08.010. 18929686 PMC2700030

[46] Kegler MC , Blumenthal DS , Akintobi TH , et al. Lessons learned from three models that use small grants for building academic-community partnerships for research. J Health Care Poor Underserved. 2016;27:527–548. doi: 10.1353/hpu.2016.0076. 27180693 PMC5554883

[47] Main DS , Felzien MC , Magid DJ , et al. A community translational research pilot grants program to facilitate community-academic partnerships: lessons from Colorado’s clinical translational science awards. Prog Community Health Partnersh. 2012;6:381–387. doi: 10.1353/cpr.2012.0036. 22982851 PMC3607296

[48] Martinez LS , Peréa FC , Ursillo A , Weeks FH , Goldstein-Gelb W , Brugge D. A democratic university-community administrative body dedicated to expanding community-engaged research: the tufts community research center (TCRC). Community Dev (Columbus, Ohio). 2013;44:97–110. doi: 10.1080/15575330.2012.678873.

[49] Rodgers KC , Akintobi T , Thompson WW , Evans D , Escoffery C , Kegler MC. A model for strengthening collaborative research capacity: illustrations from the atlanta clinical translational science institute. Health Educ Behav. 2014;41:267–274. doi: 10.1177/1090198113511815. 24311741 PMC4067460

[50] Tendulkar SA , Chu J , Opp J , et al. A funding initiative for community-based participatory research: lessons from the harvard catalyst seed grants. Prog Community Health Partnersh. 2011;5:35–44. doi: 10.1353/cpr.2011.0005. 21441667 PMC3726716

[51] Coombe CM , Simbeni S , Neal A , et al. Building the foundation for equitable and inclusive research: seed grant programs to facilitate development of diverse CBPR community–academic research partnerships. J Clin Transl Sci. 2023;7:e2. doi: 10.1017/cts.2022.495. 36755548 PMC9879886

[52] Nix M , McNamara P , Genevro J , et al. Learning collaboratives: insights and a new taxonomy from AHRQ’s two decades of experience. Health Affair. 2018;37:205–212. doi: 10.1377/hlthaff.2017.1144. 29401014

[53] Wenger E. Communities of practice: A brief introduction. University of Oregon. (https://scholarsbank.uoregon.edu/xmlui/bitstream/handle/1794/11736/A%20brief%20introduction%20to%20CoP.pdf?sequence=1&isAllowed=y) Accessed January 14, 2022.

